# Differential Sympathetic Vasomotor Activation Induced by Liver Cirrhosis in Rats

**DOI:** 10.1371/journal.pone.0152512

**Published:** 2016-04-07

**Authors:** Heder F. G. Estrela, Elaine S. Damásio, Eduardo K. U. N. Fonseca, Cássia T. Bergamaschi, Ruy R. Campos

**Affiliations:** Department of Physiology, Escola Paulista de Medicina, Federal University of São Paulo, São Paulo, Brazil; IDIBAPS - Hospital Clinic de Barcelona, SPAIN

## Abstract

We tested the hypothesis that there is a topographical sympathetic activation in rats submitted to experimental cirrhosis. Baseline renal (rSNA) and splanchnic (sSNA) sympathetic nerve activities were evaluated in anesthetized rats. In addition, we evaluated main arterial pressure (MAP), heart rate (HR), and baroreceptor reflex sensitivity (BRS). Cirrhotic Wistar rats were obtained by bile duct ligation (BDL). MAP and HR were measured in conscious rats, and cardiac BRS was assessed by changes in blood pressure induced by increasing doses of phenylephrine or sodium nitroprusside. The BRS and baseline for the control of sSNA and rSNA were also evaluated in urethane-anesthetized rats. Cirrhotic rats had increased baseline sSNA (BDL, 102 vs control, 58 spikes/s; p<0.05), but no baseline changes in the rSNA compared to controls. These data were accompanied by increased splanchnic BRS (p<0.05) and decreased cardiac (p<0.05) and renal BRS (p<0.05). Furthermore, BDL rats had reduced basal MAP (BDL, 93 vs control, 101 mmHg; p<0.05) accompanied by increased HR (BDL, 378 vs control, 356; p<0.05). Our data have shown topographical sympathetic activation in rats submitted to experimental cirrhosis. The BDL group had increased baseline sSNA, independent of dysfunction in the BRS and no changes in baseline rSNA. However, an impairment of rSNA and HR control by arterial baroreceptor was noted. We suggest that arterial baroreceptor impairment of rSNA and HR is an early marker of cardiovascular dysfunction related to liver cirrhosis and probably a major mechanism leading to sympathoexcitation in decompensated phase.

## Introduction

The cardiovascular autonomic dysfunction is well established in cirrhosis of different etiologies, with the degree of sympathetic activation strongly associated with the disease severity and poor prognosis [1, 2]. Increased sympathetic nerve activity was observed in both patients and experimental animal models of liver cirrhosis, evidenced by increased plasma levels of norepinephrine or norepinephrine spillover from the neuroeffector junctions, compatible with the activation of the sympathetic nervous system [3–10]. Although some evidences suggest that changes in sympathetic vasomotor activity are associated with the stage of liver disease, controversy exists in the literature as other studies have not found any correlation between changes in the sympathetic vasomotor tone and cirrhosis [11–15]. However, there are evidences suggesting that the changes in sympathetic vasomotor activity are related to the stage of the liver disease. Therefore, previous studies described no changes in sympathetic vasomotor tone in cirrhosis. Considering that the vasomotor sympathetic nerve activity is topographically organized [16], in the present study, we tested the hypothesis that there is a topographical sympathetic activation in rats submitted to experimental cirrhosis, renal and splanchnic sympathetic nerves were evaluated.

Abnormal cardiovascular homeostasis is also manifested by blunted responsiveness of blood vessels and attenuated reflexes such as the arterial baroreceptors [8, 17, 18]. Normally, cardiac, splanchnic and renal sympathetic nerve activities are substantially regulated by high-pressure arterial baroreceptors [19]. Previous evidences suggest that the baroreceptor control of heart rate and sympathetic nerve activity is altered in liver cirrhosis [20–24] and impairment reflex is related with the degree of hepatic dysfunction and mortality [1, 25]. Thus, impairment of baroreceptor sensitivity may trigger sympathetic activation in cirrhosis. Therefore, the second aim of the present study was to evaluate the baroreceptor function in rats submitted to an experimental model of cirrhosis induced by bile duct ligation (BDL), a model regularly employed to investigate cirrhosis [26–28].

## Methods

### Animals and ethical approval

Male Wistar rats weighing 200 to 250 g obtained from the Federal University of São Paulo were housed in group cages (n = 5) in a room with controlled temperature, a 12:12h light-dark cycle and free access to water and food. Experiments followed the Guiding Principles in the Care and Use of Animals of the American Physiological Society and were approved by the Institutional Review Board of the Federal University of Sao Paulo (protocol 1095/10).

### Experimental design

The rats were distributed into two experimental groups: control and bile duct ligation (BDL). The present study was divided into two independent series of experiments. In the first series, the hepatic function was assessed in the two groups of rats. In the second series of experiments, the cardiovascular parameters: mean arterial pressure (MAP), heart rate (HR), and arterial baroreceptor reflex control of HR were assessed in conscious rats. At least 24h after that, the same group of rats was slowly anesthetized with urethane (1.4 g/kg, iv) (Sigma-Aldrich, St Louis, MO) to avoid any change in MAP, and the basaline splanchnic (sSNA) and renal sympathetic nerve activity (rSNA) were recorded. The baroreceptor function for the control of sSNA and rSNA were also evaluated.

### Experimental cirrhosis

Cirrhosis was induced in the rats by the bile duct-ligation rat model, a well-established model of cirrhosis, portal hypertension, hemodynamic and pharmacological studies [29–31]. Animals were anesthetized with ketamine (40 mg/kg body weight i.p.) and xylazine (20 mg/kg body weight i.p.) (Vetbrands, Jacarei, Brazil). The BDL was performed as previously described [32]. Briefly, the common bile duct was exposed by median laparotomy, doubly ligated and resected between the two ligations. All the functional analysis and parameters measurement were performed 4 weeks after bile duct ligation. This delay is necessary for the development of secondary biliary cirrhosis [29].

### Biochemical analysis of serum

The liver function parameters, alkaline phosphatase (AP), alanine aminotransferase (ALT) and aspartate aminotransferase (AST), were measured by colorimetric assay employing commercial kits (Labtest Diagnostic AS, Belo Horizonte, Brazil).

### Analysis of cardiovascular parameters and cardiac baroreceptor reflex sensitivity (BRS) in conscious rats

For intravenous injection of drugs and direct arterial pressure recording, the rats were anesthetized with ketamine and xylazine (40 and 20 mg/kg, IP, respectively) and catheterized with femoral venous and arterial catheters. Following ≥24h of surgical recovery, MAP and HR were recorded in conscious rats using an analog-digital board that communicated with the PowerLab software (ADInstruments, Sydney, Australia). Subsequently, pressor doses of phenylephrine (3, 6, and 10 μg/kg, iv) and depressor doses of sodium nitroprusside (7, 17, and 20 μg/kg, iv) (Sigma-Aldrich) were acutely administered to induce a blood pressure increase or decrease and reflex bradycardia or tachycardia, respectively. The vasoactive drugs were administered with ≥5 min intervals between doses, until blood pressure returned to baseline. Values of matching MAP variations (ΔMAP from 20 to 55 mmHg) with reflex HR (ΔHR) response were separately plotted for each vasoactive drug to create linear regression curves of baroreceptor function for each group, and their slopes (beats/min/mmHg) were compared to test changes in the baroreceptor reflex sensitivity.

### Analysis of autonomic parameters and splanchnic and renal baroreceptor reflex sensitivity in urethane-anesthetized rats

Rats were slowly anesthetized with urethane (1.4 g/kg, iv). For the renal sympathetic nerve activity (rSNA) and splanchnic sympathetic nerve activity (sSNA) recordings, the left renal and splanchnic nerve were retroperitoneally exposed, placed on bipolar silver electrodes and once the conditions for nerve recording was established, the nerve and electrode were covered with paraffin oil. The signal from the renal nerve was displayed on an oscilloscope (TDS 220; Tektronix, Portland, OR), and the nerve activity was amplified (gain 20 K, Neurolog; Digitimer, Welwyn Garden City, UK), filtered by a band-pass filter (100–1,000 Hz), and collected. Data analysis was then performed using the PowerLab data acquisition system (ADInstruments, Australia). At the end of the experiments, the background noise level of SNA was determined following hexamethonium bromide administration (30 mg/kg, iv) (Sigma-Aldrich). The sSNA and rSNA were rectified online, integrated from the raw data obtained for each heart period, and expressed as volts-seconds (V.s). In addition, the neural activity was analyzed offline using the appropriate software (Spike Histogram; ADInstruments, Australia). For this purpose, the raw nerve signal was passed through a spike discriminator (PowerLab; ADInstruments, Australia) to remove background noise. The total nerve activity expressed in spikes per second (spikes/s) was then computed from the time at which nerve activity changed from the basal value to when it returned to the basal value. The baseline sSNA and rSNA are expressed as spikes per second (spikes/s) over a period of 60s. The mean values obtained were compared to the mean values determined before each test, as reported previously [33]. Only the experiments in which the level of background noise was confirmed at the end of the experiments following hexamethonium and terminal anesthesia are included in this report. For the analysis of arterial baroreceptor control of sSNA and rSNA, blood pressure was altered by ramp infusion of phenylephrine (100 μg/ml, 6 ml/h, iv) or sodium nitroprusside (200 μg/ml, 6 ml/h, iv) to raise or lower blood pressure by approximately 40 mmHg for 60 seconds, respectively. During infusions, each rat received no more than 0.1 ml. Phenylephrine and sodium nitroprusside were infused with a 3 ml syringe mounted on a syringe pump (KdScientific, USA) [34]. The barorreflex sensitivity was evaluated by the reflex changes on rSNA and sSNA every 5 mmHg of MAP change (from 5 to 40 mmHg). The slope analysis represents the baroreflex gain (ΔrSNA/ΔMAP) from each individual animal and was expressed as pps/mmHg.

### Statistical analyses

Results were expressed as mean ± standard error of the mean (SEM). Data were evaluated by unpaired Student’s t-test using Graph-Pad Prism (San Diego, CA, USA). The level of statistical significance was defined as P<0.05.

## Results

Table 1 shows the data for body, liver and kidney weights, organ weight-to-body weight ratio, and serum alkaline phosphatase, aspartate aminotransferase and alanine aminotransferase level in the control and BDL groups. BDL rats had significantly increased values for liver and kidney weight, liver/body weight and kidney/body weight compared to control. Furthermore, serum transaminases levels were significantly increased in the cirrhotic rats. However, the cirrhotic rats had lower weight gain compared to control group.

**Table 1:** Body and organ weights, organ weight-to-body weight ratio, and liver function parameters in control and bile duct ligation (BDL) experimental groups.

	Control	BDL
**Body wt, g (n = 9)**	**331 ± 3**	**292 ± 4** *
**Liver wt, g (n = 6)**	**16 ± 1**	**24 ± 2** *
**Liver wt/body wt, g (n = 6)**	**0.05 ± 0.004**	**0.08 ± 0.005** *
**Kidney wt, g (n = 6)**	**1.62 ± 0.04**	**1.90 ± 0.10** *
**Kidney wt/body wt, g (n = 6)**	**0.0052 ± 0.0001**	**0.0066 ± 0.0003** *
**AP, U/L (n = 5)**	**47 ± 4**	**99 ± 18** *
**ALT, U/L (n = 4)**	**60 ± 6**	**210 ± 44** *
**AST, U/L (n = 4)**	**198 ± 24**	**566 ± 97** *

Values are expressed as mean ± SEM.

wt, weight;

n, number of rats;

BDL, bile duct ligation;

AP, alkaline phosphatase;

AST, aspartate aminotransferase;

ALT, alanine aminotransferase.

* P<0.05 compared to control group (Student’s t test).

### Baseline cardiovascular parameters and cardiac baroreceptor reflex sensitivity in conscious rats

Under baseline conditions, HR was significantly higher and MAP significantly lower in the cirrhotic rats compared to control rats (Table 2).

**Table 2:** Summary of data on cardiovascular parameters in the different experimental groups.

	Control (n = 9)	BDL (n = 9)
**MAP, mmHg**	**101 ± 1**	**93 ± 1** *
**HR, beats/mim**	**356 ± 3**	**378 ± 4** *

Values are expressed as the mean ± SEM.

n, number of rats;

BDL, bile duct ligation;

MAP, mean arterial pressure;

HR, heart rate.

* P < 0.05, compared to control group (Student’s t test).

**Fig 1:**
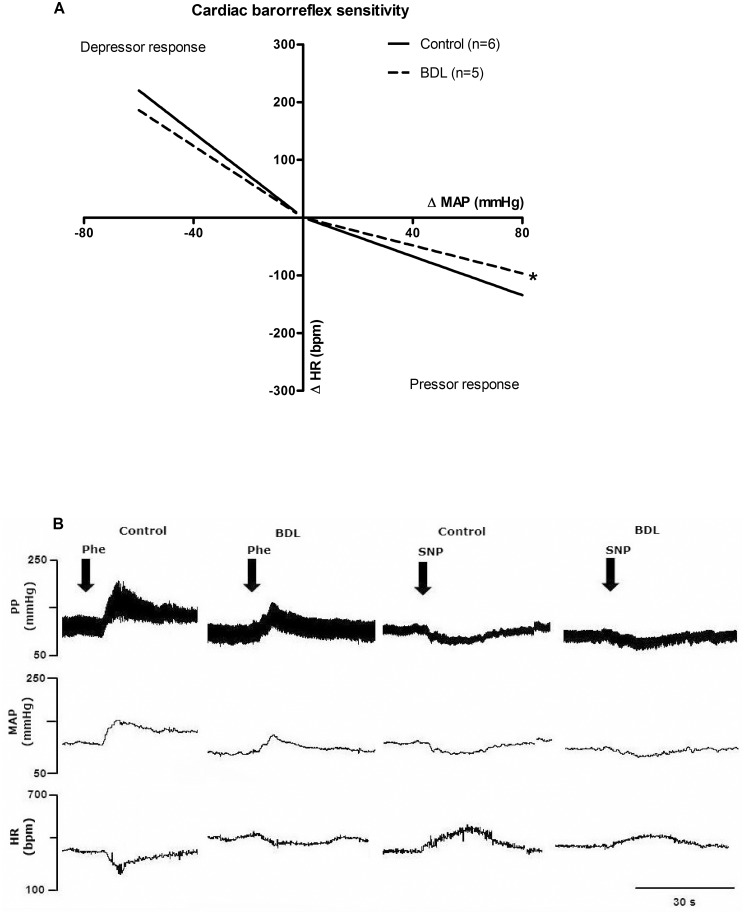
Baroreceptor reflex sensitivity for heart rate (HR). (**A**) Linear regression graph showing: depressor response—the reflexive increase in heart rate (ΔHR) to decrease the mean arterial pressure (ΔMAP) produced by acute administration of sodium nitroprusside (7, 17, and 20 μg/kg); and pressor response—HR decrease reflex response to increase MAP produced by acute administration of phenylephrine (3, 6, and 10 μg/kg) in the control and bile duction ligation (BDL) groups. n, number of rats. *P < 0.05 compared to control group (Sutdent’t test). (**B**) Representative recording of cardiovascular responses induced by acute infusion of phenylephrine (Phe) and sodium nitroprusside (SNP) in the control and BDL groups. Pulse pressure (PP), heart rate (HR), beats per minute (bpm) and mean arterial pressure (MAP).

The BRS was attenuated in conscious cirrhotic rats (Fig 1 and Table 3).

**Table 3:** Slope values obtained from the linear regression lines in the control or BDL groups.

Groups	Cardiac reflex	
	Pressor response	Depressor response
	Slope (sps/mmHg)	Slope (sps/mmHg)
**Control (n = 6)**	**-1.68 ± 0.12**	**-3.43 ± 0.32**
**BDL (n = 5)**	**-1.21 ± 0.14** *	**-3.21 ± 0.36**

Values are expressed as the mean ± SEM.

n, number of rats,

sps, spikes per second;

BDL, bile duct ligation.

*P < 0.05 compared to control group (Student’s t test).

### Baseline autonomic parameters and splanchnic and renal barorreflex sensitivity in urethane-anesthetized rats

**Fig 2:**
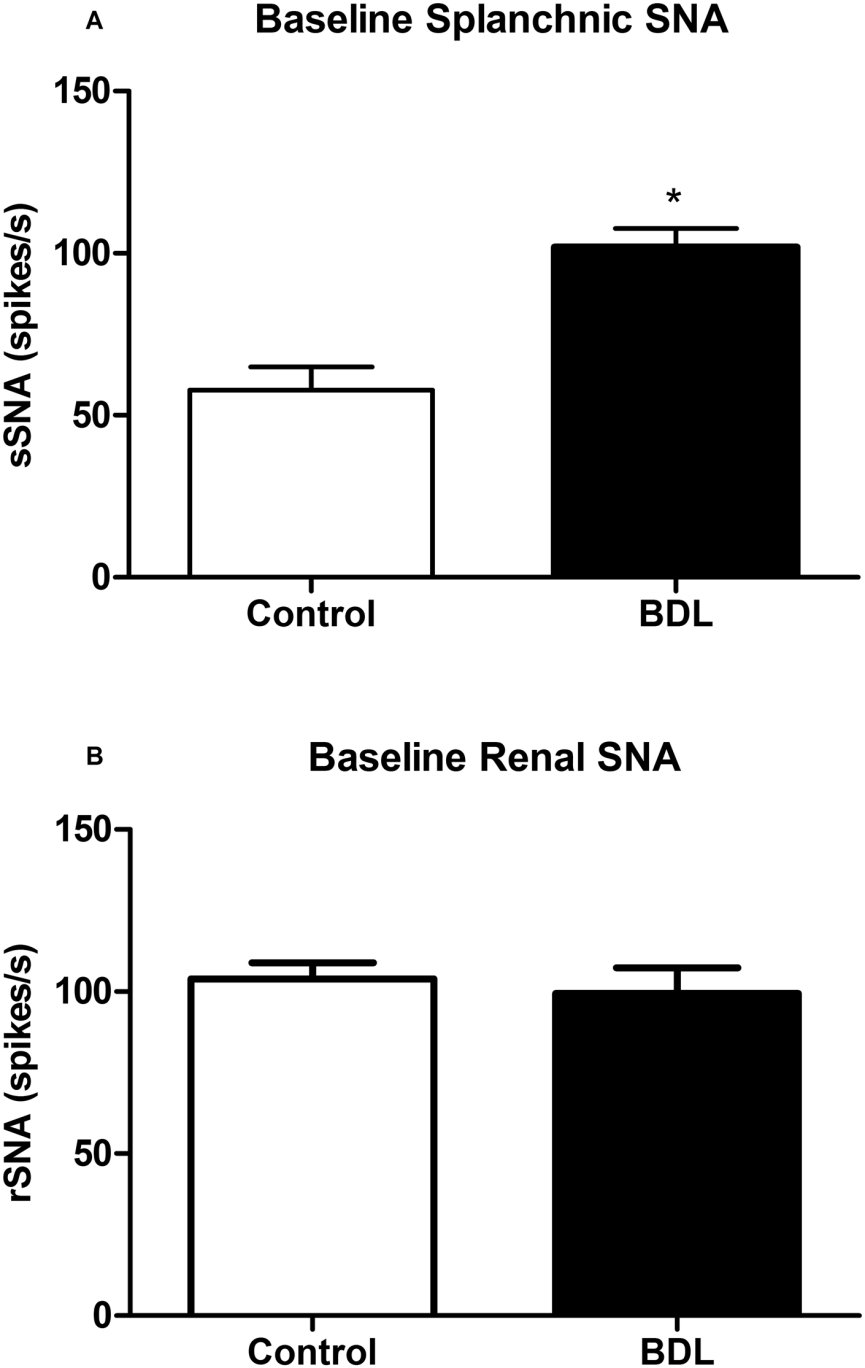
Baseline sympathetic nerve activity. (**A**) Splanchnic sympathetic nerve activity (sSNA, n = 9) and (**B**) renal sympathetic nerve activity (rSNA, n = 9) at baseline level in the different groups. BDL, bile duct ligation. Error bars indicate SEM. *p < 0.05 compared to control group (Student’s t test).

BDL group had higher baseline sSNA than control (BDL, 102 ± 6, n = 9 vs control, 58 ± 7 spikes/s, n = 9; p<0.05). However, no difference was found between control and BDL groups in baseline rSNA (BDL, 99 ± 8, n = 9 vs control, 104 ± 5, n = 9), as shown in Fig 2.

**Fig 3:**
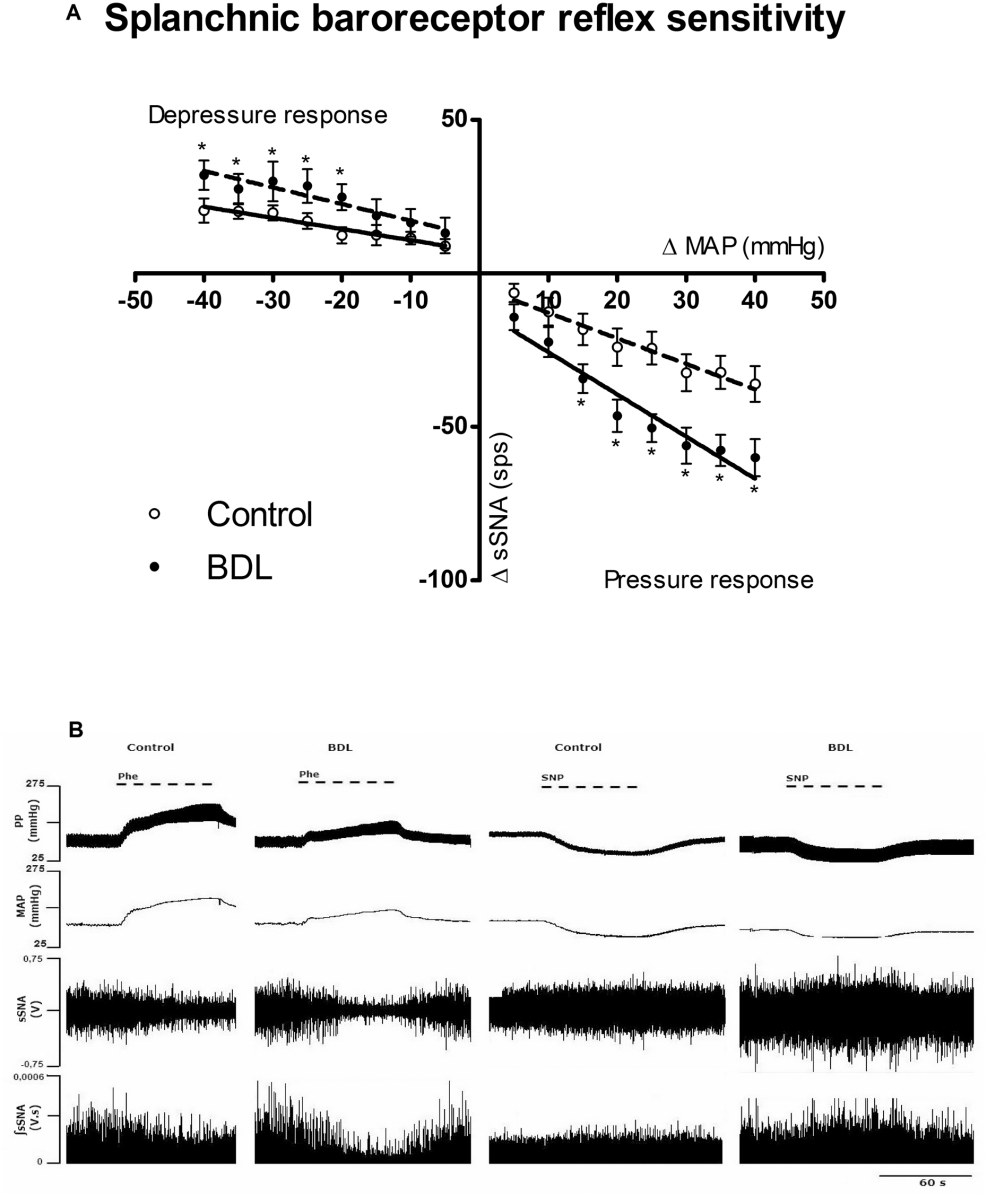
Baroreceptor reflex sensitivity for splanchnic sympathetic nerve activity (sSNA). (**A**) Linear regression graph showing: depressor response—the reflexive increase in sSNA to decrease the mean arterial pressure (MAP) produced by ramp infusion of sodium nitroprusside (200μg/ml, 6ml/h, iv); and pressor response—sSNA decrease reflex response to increase MAP produced by ramp infusion of phenylephrine (100μg/ml, 6ml/h, iv) for 60s in the control and bile duction ligation (BDL) groups. n, number of rats *P < 0.05 compared to control group (Sutdent’*t* test). (**B**) Representative recording cardiovascular responses induced by ramp infusion of phenylephrine (Phe) and sodium nitroprusside (SNP) in the control and BDL groups. Pulse pressure (PP), mean arterial pressure (MAP), sSNA expressed in volts (V) and integrated (ʃ) sSNA expressed in volts-seconds (V.s).

**Fig 4:**
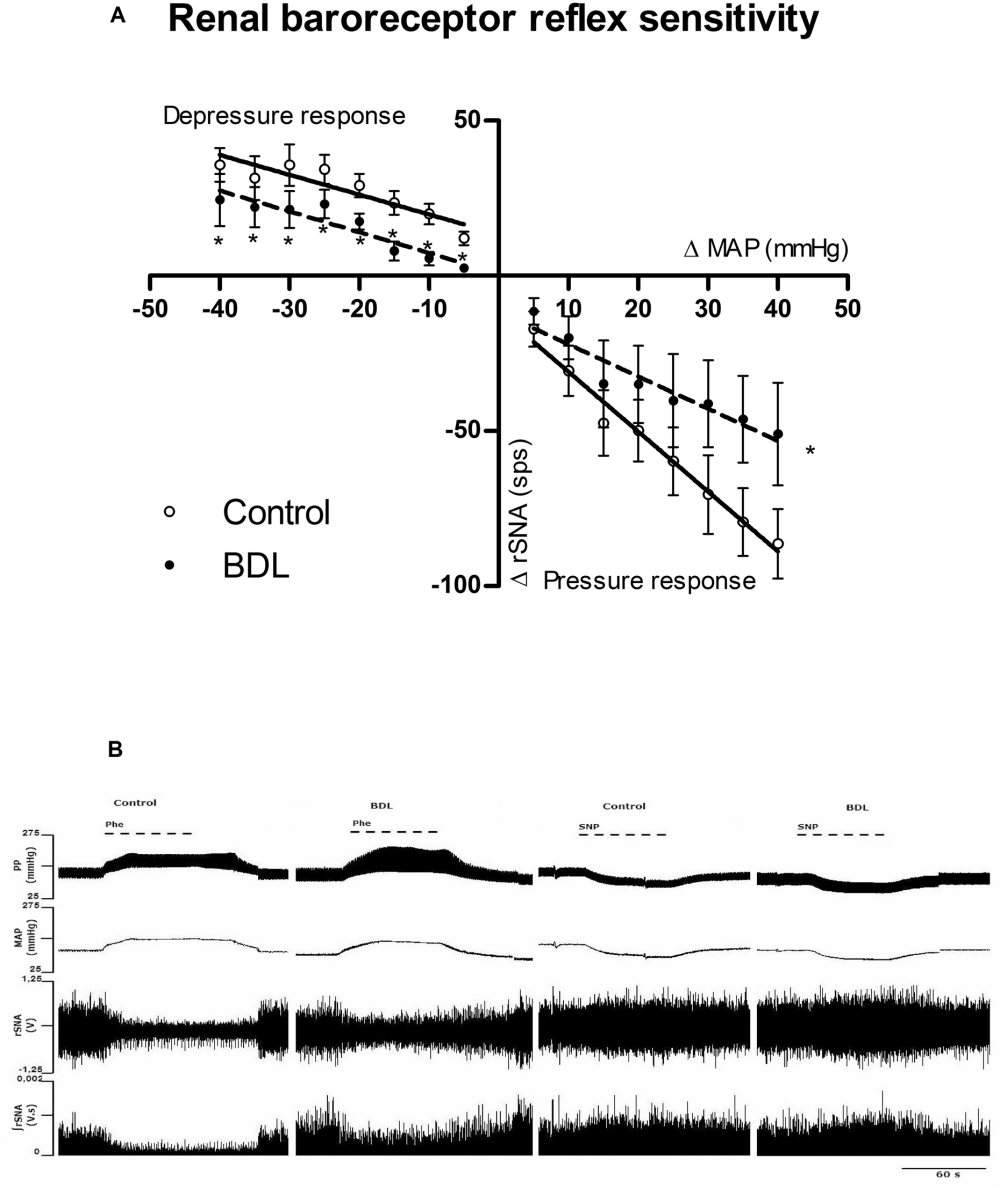
Baroreceptor reflex sensitivity for renal sympathetic nerve activity (rSNA). (**A**) Linear regression graph showing: depressor response—the reflexive increase in rSNA to decrease the mean arterial pressure (MAP) produced by ramp infusion of sodium nitroprusside (200μg/ml, 6ml/h, iv); and pressor response—rSNA decrease reflex response to increase MAP produced by ramp infusion of phenylephrine (100μg/ml, 6ml/h, iv) for 60 seconds in the control and bile duction ligation (BDL) groups. n, number of rats *P < 0.05 compared to control group (Sutdent’t test). (**B**) Representative recording cardiovascular and autonomic responses induced by ramp infusion of phenylephrine (Phe) and sodium nitroprusside (SNP) in the control and BDL groups. Pulse pressure (PP), mean arterial pressure (MAP), rSNA expressed in volts (V) and integrated (ʃ) rSNA expressed in volts-seconds (V.s).

Fig 3 shows the splanchnic baroreceptor reflex sensitivity in response to phenylephrine and sodium nitroprusside ramp infusion. BDL rats had increased splanchnic BRS. The slope values (Table 4) were significantly higher in BDL compared to the controls. However, when the BRS for rSNA was compared between groups, a significant impairment was observed in BDL group (Fig 4). The individual slope values are shown in Table 4.

**Table 4:** Slope values obtained from the linear regression lines in the groups studied.

Groups	Splanchnic SNA		Renal SNA	
	Pressor response	Depressor response	Pressor response	Depressor response
	Slope (sps/mmHg)	Slope (sps/mmHg)	Slope (sps/mmHg)	Slope (sps/mmHg)
**Control**	-0.83 ± 0.14 (n = 9)	-0.45 ± 0.07 (n = 9)	-1.96 ± 0.22 (n = 9)	-0.60 ± 0.18 (n = 9)
**BDL**	-1.60 ± 0.25 (n = 9) *	-0.77 ± 0.12 (n = 9) *	-0.94 ± 0.26 (n = 7) *	-0.62 ± 0.13 (n = 7)

Values are expressed as the mean ± SEM.

n, number of rats,

sps, spikes per second;

BDL, bile duct ligation.

*P < 0.05 compared to control group (Student’s t test).

## Discussion

The major new findings of the present study were 1) the preferential increase in sSNA in BDL rats, but not in rSNA, 2) the enhanced BRS for sSNA and decreased for HR and rSNA. The autonomic changes in BDL rats were accompanied by a significant drop in MAP and increased HR.

Four weeks after BDL, the rats had confirmed liver cirrhosis characterized by elevated serum transaminases levels and hepatomegaly, but without ascites. The reduced MAP observed in cirrhotic rats was probably a result of systemic vasodilation that leads to low systemic vascular resistance as a consequence of a combination of overproduction of circulating vasodilators, vasodilators of intestinal or systemic origin, vasodilators that escape degradation in the diseased liver or bypass the liver through portosystemic collaterals, reduced resistance to vasoconstrictors and increased sensitivity to vasodilators [35, 36].

The autonomic dysfunction, characterized by a sympathovagal imbalance is probably a cause of elevated basal HR in BDL rats, considering that increased cardiac SNA, accompanied by decreased vagal activity was described in cirrhosis [37]. Increased baseline sSNA is consistent with previous studies that found elevated norepinephrine levels in portosystem and mesenteric vessels from patients [3, 5] and in experimental models of cirrhosis [38, 39] suggesting increased sSNA activity. Furthermore, it has been recently reported that cirrhotic rats display almost doubled and about 30% increased NA release caused by first and subsequent electric sympathetic perivascular nerve stimulation, around superior mesenteric artery, respectively [39].

The present study confirmed the increased sSNA by directly recording the splanchnic nerve activity. However, the baseline rSNA was not changed by BDL. These data is consistent with Henriksen et al. (1989) that found elevated norepinephrine in the liver and intestine, but no in renal vein from preascitic cirrhotic patients, suggesting specific sympathetic hypertonia to splanchnic territory in this phase [40]. However, several previous studies reported sympathetic hyperactivity to the kidneys in liver cirrhosis [4, 6–8, 41, 42]. This discrepancy is probably related to the phase of liver disease chosen for the previous studies. It is well known that Wistar rats are in preascitic phase or compensated liver cirrhosis in the first 2–4 weeks following BDL. Only 5 or more weeks after BDL the rats suffer with decompensated liver cirrhosis and ascites [29, 43]. Although, in the present study, the BDL group had impairment renal BRS, that was not sufficient to increase baseline rSNA in animals with compensated cirrhosis. We found at this stage of the disease an increase in the urinary volume. However, previous studies described that 4 weeks after BDL, there is no important histological changes in the kidneys. However, it was reported 4 weeks after BDL a significant increase in serum creatinine but under values of normality (1.2 mg/dl). Six weeks after BDL the serum creatinine increased to 2.5 mg/dl, showing kidney function impairment [44].

Despite the fact that the topographical sympathetic change is organized in BDL rats is not well established, several brain regions have been implicated in the generation of differential sympathetic outflow to distinct cardiovascular targets [16, 45, 46], including the periaqueductal gray [47, 48], nucleus tractus solitaries [49], paraventricular nucleus of the hypothalamus [50, 51] and the rostral ventrolateral medulla (RVLM) [52–54]. Although the idea of a viscerotopic organization of sympathetic outflow within the RVLM has been fairly well established in cats [52, 53, 55–57], studies performed with rats have suggested a control of regional SNA by the RVLM [58, 59]. Therefore, it is plausible to suggest that changes in neurotransmission in brain regions such as the RVLM, occur in rats with cirrhosis, leading to a preferential or specific changes in efferent sympathetic nerve discharge to different targets.

A large number of chemo and mechanic receptor are present in the liver that sense ions, nutrients and blood flow and send signals to the brain [60]. Our hypothesis is that at this stage of the liver disease, increased activity of afferents from the liver to the central nucleus of autonomic cardiovascular control are responsible for the autonomic changes observed in the present study. At this stage of liver disease increased splanchnic SNA may be a compensatory mechanism in response to the significantly reduced splanchnic vascular resistance that leads to increased splanchnic blood flow and portal pressure [18, 35, 36]. However, there are evidences that prolonged adrenergic tissue stimulation leads to diminished responsiveness to subsequent activation by catecholamines, aggravating the splanchnic vasodilation in liver cirrhosis [39]. Furthermore, in humans, intrahepatic efferent nerves terminate at Disse’s space in close proximity to hepatic stellate cells (HSC), sinusoidal endothelial cells (SEC) and hepatocytes [61, 62]. Sympathetic release of noradrenalin and substance P (SP) causes contraction of the liver sinusoids [60]. These molecules are thought to act on HSC and SEC inducing the contraction of the sinusoids [63], aggravating portal hypertension. In addition, sympathoexcitation to the liver inhibits hepatic oval cells (HOC) actions and activates HSC [60, 64], accelerating fibrogenic process.

The efferent SNA control by arterial baroreceptors also presented topographic differences in BDL group. Cardiac e renal BRS were reduced and splanchnic BRS was increased. Several studies described impairment of cardiac and renal BRS in patients as well as in experimental models of cirrhosis [8, 20, 21, 23, 24].

Despite the fact that components of the renin-angiotensin system (RAS) were not measured in the present study, there is an inverse relationship between the BRS and RAS in liver disease, which may be involved in reducing the BRS [24], decreasing sympathetic and parasympathetic reflex responses to blood pressure changes [25]. In fact, Thiesson et al. (2007) observed that rats with compensated cirrhosis presented increased plasma renin concentration [43]. Furthermore, several studies showed that treatment of cirrhotic animals with angiotensin II antagonists promotes an improvement or normalization of arterial baroreceptor reflex sensitivity [2, 65].

There is substantial evidence that the RAS is deeply involved in the impaired baroreflex function in animals or patients with cirrhosis and that the reflex control of circulation may be partially restored by blockade of RAS [24, 66]. We suggest that the arterial baroreceptor impairment of rSNA and HR is an early marker of cardiovascular dysfunction related to liver cirrhosis and probably a major mechanism leading to sympathoexcitation in decompensated late phase. It has been described that impairment of baroreceptor function could increase the severity of diseases such as arterial hypertension and heart failure [67] and we described in the present study a similar mechanism in experimental cirrhosis for the control of renal sympathetic activity. In humans, it has been reported that there is a close correlation between baroreceptor sensitivity impairment and the severity of cirrhosis [23, 24].

However, the splanchnic sympathoexcitation in BDL rats was independent of arterial baroreceptor reflex dysfunction. Considering that the splanchnic vascular bed receives 25% to 30% of the total cardiac output and has a major role in blood storage [68], an increase in sympathetic tone to its arterioles leads to a significant rise in total peripheral resistance and an increase in sympathetic tone to its small veins and venules, resulting in a significant mobilization of blood to the central veins and an enhanced cardiac output following a rise in right atrial filling. The combined effect leads to a redistribution of blood volume from the splanchnic venous circulation to the systemic arterial vascular compartment, causing a rise in arterial pressure [69].

Although we did not investigate the venous congestion in BDL rats, we believe that increased sSNA activity is a compensatory mechanism to reduce venous congestion in liver disease, and mobilize blood volume from splanchnic region to other body tissues. In conclusion, our data have shown topographical sympathetic activation in rats submitted to experimental cirrhosis.

## Conclusion

The BDL group had increased baseline sSNA, independent of arterial baroreceptor reflex dysfunction and no changes in baseline rSNA, although impairment of rSNA and HR control by arterial baroreceptor was found. The changes are probably organized by central nuclei of the brain related to autonomic cardiovascular control. However, the mechanisms underlying increased SNA and BRS dysfunction in cirrhosis is still not well understood.

### Perspectives

New studies in late decompensate cirrhosis would be important to further our understanding regarding the evolution of autonomic and kidney dysfunction as well as the relationship of increased sympathetic drive to splanchnic territory and portal hypertension. The reno-renal and the hepato-renal reflex assessing during cirrhosis development and how they interact with the arterial baroreceptor reflex could contribute to elucidate the mechanisms leading to autonomic dysfunction in cirrhosis.
